# Book Reviews

**Published:** 1832-03

**Authors:** 


### BIBLIOGRAPHICAL NOTICES.

*A Modern Anatomy of the Human Spleen; to which are added, some Strictures on* Sir Anthony Carlisle's *Theory respecting the Functions of that Organ.* By P. Mac Nolty, Surgeon, &c.—8vo. pp. 71. Renshaw and Rush, London.

Like a lady's letter, the postscript much exceeds the body of this pamphlet, not however in importance, but in length; and we could have been well content if the Strictures, spreading over about fifty pages, had been occupied by the more legitimate investigation of the Anatomy of the Spleen, to which only seventeen pages have been devoted, instead of thus dedicating three fourths of a very small book to a vituperative tirade, foreign to the subject, and discreditable to a work on science. Mr. Mac Nolty appears to be a man of observation and ingenuity, and we trust that, when the sequel to the present dissertation is published, he will refrain from travelling so far out of the record, and give to his purchasers what the title promises.

The object of the seventeen pages, on which alone we shall offer any comment, is to shew that the structure of the spleen is vascular, that its vessels are tortuous, and greatly sacculate, so as to give them a varicose appearance, which has deceived previous anatomists into the opinion that this viscus is cellular. But the author has only *said*; he has not, at least in this work, proved his assertion, although we confess we are not inclined to doubt its truth; and his pamphlet would have been greatly increased in value had the fifty pages, which we think wasted, been occupied in a demonstration of the structure adverted to.

As to the physiology, which is only slightly noticed, we will not decide as to the priority of the idea between so many claimants for the doctrine of the diverticular functions of the spleen: we, in our number for May 1831, gave a copious account of them all, in a review of "HOLLAND on the Physiology of the Fœtus, Liver, and Spleen," and to this we refer our readers. Mr. Mac Nolty chiefly differs from other authors in believing the functions of the spleen and thyroid gland not to be similar. We shall look with anxiety for his more elaborate treatise, and doubt not, from the energy displayed in this, although we think in part misdirected, that the forthcoming volume will be more worthy of its author and useful to his profession.

Should this pamphlet be reprinted, we would recommend the publisher to look more strictly to its typographical execution; for, although classical quotations are profusely scattered on almost every page, they are not too correct in the transcript; and we like quantity far less than quality.

---

*An Atlas of Surgical Apparatus; being a Series of Delineations of the most important Mechanical Auxiliaries of Surgery, with descriptive Letter-press, explaining their several Uses and Modes of Application.* By H. T. CHAPMAN, Member of the Royal College of Surgeons, and late House Surgeon to St. Bartholomew's Hospital.—Highley, London.

*A Brief Description of Surgical Apparatus: intended to accompany a Series of Delineations of the most important Mechanical Auxiliaries of Surgery.* By H. T. CHAPMAN, Member of the Royal College of Surgeons, and late House Surgeon to St. Bartholomew's Hospital.—8vo. pp. 115. Highley, London.

IN undertaking the present work, the author has been chiefly influenced by a recollection of the difficulties experienced by himself, and many of his fellow-stndents, when first entering upon the management of surgical cases in which mechanical assistance was required, in consequence of a very imperfect acquaintance with the scattered resources of this branch of surgery: and he truly remarks, that this knowledge is important, independently of its intrinsic value; inasmuch as the reputation of a young surgeon is more frequently founded upon address displayed even in the most common manœuvres of this department, than in the possession of more solid but less conspicuous qnalifications; for, as every looker-on can appreciate the manner of their execution, they are made the standard by which his general ability is estimated. The public hospitals are the only places where these operations can be repeated with sufficient frequency to secure dexterity, and most students who have "walked" either of these establishments have no opportunity of improving themselves in this important branch of their surgical education. "In the continental schools, besides hospital practice, students have other opportunities of exercising themselves; they may resort to the public course of lectures, one of which is devoted to surgical apparatus, to look over the several articles, see them applied, and afterwards practise their application, upon a man paid to attend for that purpose; or a party may enter into an arrangement for mutually applying them upon each other, taking one of the several works on the subject to be met with in the French and German languages."

In this country we do not possess a collective detail of the surgical mechanism in use at the present day, and Mr. CHAPMAN "attempts" (we would say, succeeds,) in the present publications to supply the deficiency. To render his work more complete, he has drawn from the best English and French surgical authorities whatever information was necessary to his subjects. The drawings have not been copied from the delineations of others, but, with very few exceptions, have been taken from the different apparatus when actually applied to the subject. As the surgeon in private practice cannot be expected to provide himself with the *armamentarium chirurgicum* of a hospital, a variety of simple and domestic contrivances, which can be readily procured, have been introduced as substitutes for some of the more expensive forms of apparatus.

In the "Brief Description," an account is given of bandages of all kinds; apparatus for fractures and for reducing dislocations; and of surgical instruments, whether for the performance of operations or for other purposes.

The "Atlas" contains twenty well-executed plates, representing every kind of bandages, when applied in different injuries and operations; apparatus for fractures of the limbs; apparatus for reducing dislocations; and surgical instruments.

To surgical students, and even practitioners, this work will be found of the greatest assistance.

It is the intention of Mr. Chapman to undertake a course of practical demonstrations of bandages and surgical apparatus in the neighbourhood of any of the London medical schools, as soon as a class of fifteen shall be formed. The object of these demonstrations will be not merely to exhibit an assemblage of the various articles of surgical apparatus, but also to offer the means of practical exercise in their application, by engaging the attendance of a person upon whom the bandages, apparatus for fractures, for the reduction of dislocations, &c. will be adjusted by each student in rotation. Such a course of lectures is much wanted in the metropolis, and we cannot doubt that Mr. Chapman will speedily obtain many more pupils than he requires to begin his very useful undertaking.

---

*An Account of the Beulah Saline Spa at Norwood, Surrey: containing a Description of its Medicinal Properties and Effects, of the Diseases in which it is Remedial, and Directions for its Use.* By GEO. HUME WEATHERHEAD, M.D. &c.—8vo. pp. 40. Hatchard and Son, London.

WE doubt not that the Beulah spring may be as hygeian as any of its more famed fellow-candidates for public favor; and we doubt not that St. Chad's well in Gray's-inn lane, and the "Cold bath" in the fields (now fields no longer,) which receive from it a name, are quite as good as it. Indeed, the latter has some points of preference; for, in its immediate neighbourhood, by consummate mechanical skill, healthful exercise is forced upon the idle: exercise as conducive both to moral and physical reform as the basilisk which once was enveloped in a ball. But, with all their virtues, mean homely springs are too near to be valued; distance is necessary for the water-nymphs to be seen in all their imposing beauties and dazzling proportions. Things which are to be dearly bought, the proverb tells us, must far be sought.

To such, however, as hard fate forbids to wander in quest of health beyond the limits of the twopenny post, Dr. WEATHERHEAD promises as much from the streams of Beulah as other historians promise for Cheltenham, Leamington, or Bath, the Sandrock spring of the Isle of Wight, or the fetid waters of Harrowgate. Hear an extract from this chronicle.

The Beulah Spa is "especially beneficial in all cases that depend on obscure inflammatory action going on in some internal organ, where, by liberating the circulating system from some forced influence, and opening the emunctories, it should disable the local morbid action from continuing latently to prey on the vitals. It is in this manner that a course of the Beulah waters proves remedial in the following diseases: and first of Dyspepsia, or Indigestion; 2d, of Liver and Bilious Complaints; 3d, of Jaundice; 4th, of Chlorosis; 5th, of Hypochondriasis; 6th, of Chronic and Strumous Ophthalmia; 7th, of Cutaneous Diseases; 8th, Scrofula, &c. besides an unhealthy condition of the Humours, and many other disorders too numerous to mention."

What more need be said? Nothing. Perhaps, indeed, we have already said too much.

